# Chronobiological Effects on Mountain Biking Performance

**DOI:** 10.3390/ijerph17186458

**Published:** 2020-09-04

**Authors:** António Silveira, Francisco Alves, Ana M. Teixeira, Luís Rama

**Affiliations:** 1 Research Center for Sport and Physical Activity (CIDAF), Faculty of Sports Science, University of Coimbra, 3004-531 Coimbra, Portugal; ateixeira@fcdef.uc.pt (A.M.T.); luisrama@fcdef.uc.pt (L.R.); 2 Interdisciplinary Centre for the Study of Human Performance (CIPER), Faculty of Human Kinetics, University of Lisboa, 1495-751 Cruz Quebrada, Portugal; falves@fmh.ulisboa.pt

**Keywords:** circadian rhythm, cycling, MTB, mechanical power, heart rate

## Abstract

Background: the aim of this study was to analyze the chronobiology influence on the mechanical, kinematic, and physiological variables in a mountain bike (MTB) time trial. Methods: 16 mountain bike (MTB) male athletes volunteered to participate. Their characteristics were as follows: body mass 70.2 ± 5.4 kg, stature 172.7 ± 4.0 cm, body fat 9.8 ± 3.5%, and VO_2max_ 52.3 ± 3.9 mL/kg/min. Two 20 min MTB maximal protocols were applied, the first one in the morning and a second one in the afternoon period. Results: No differences were found for all the variables studied, except for the pedaling cadence (stroke rate), which showed higher values during the morning protocol (85.06 ± 7.58 vs. 82.63 ± 7.41 rpm; *p* = 0.044). Significant correlations between morning and afternoon physiological and mechanical variables were observed: heart rate (*r* = 0.871); external mechanical power—maximum (*r* = 0.845), mean (*r* = 0.938), and relative (*r* = 0.933), as well as in the cadence—stroke rate (*r* = 0.825). Conclusions: our results reveal a similar impact and significant relationship between morning and afternoon impact concerning the majority of the physiological and mechanical variables, which indicates that the period of the day does not influence the external and internal impact associated with the MTB time trial maximal protocol.

## 1. Introduction

The increased knowledge of chronobiology is crucial for the rigorous planning of training loads to optimize physical and sporting performance [1].

Atkinson and Reilly [2] define chronobiology as the science that focuses on physiological changes relative to time. The biological rhythm should be understood through the physiological changes that repeat themselves regularly in time, order, and interval [2].

The suprachiasmatic nucleus controls coordinate the rhythms of biological clocks through complex biological determinisms, such as the hormonal secretion, neural activation, heart rate frequency, and body temperature changes [1,3,4,5]. The circadian rhythm concept reports to the changes of physiological variables that occur in the organism during a whole day [2,6]. The knowledge of the circadian rhythms existence and its implications are fundamental in the optimization of sport performance, assuming that different impacts on the physiologic systems, related to the time of physical exertion, could occur [1,2,7,8].

Several studies that analyze the dynamics of the circadian rhythms in cycling have been inconclusive. Some concluded that, in cycling, the performance is not affected by the period of the day [9,10,11,12,13,14,15,16,17]. Others have observed significant differences, for similar exertion, on several variables measured at different times of the day [18,19,20,21,22,23,24,25,26]. These results demonstrate the complexity of the study, due to the diverse, depending on lifestyle, objectives for training and competition, type and schedules of planning and periodization, genetics, and other individual characteristics of each athlete; all of them affecting the athlete’s performance.

In cycling, chronobiology studies in mountain bike (MTB) with athletes using ecological field protocols are scarce. This study aims to contribute to increase the knowledge on the circadian effect in MTB tasks, using specific protocols conducted in the field, which ecologically reproduce or at least, are close to the training and competition environment.

The main objectives of this study were to compare the effects of a specific continuous maximal protocol of 20 min, performed in the morning (am) or in the afternoon (pm) on different mechanical and physiological variables.

## 2. Materials and Methods

### 2.1. Sample

Sixteen cycling male practitioners of the MTB specialty, aged 34.81 ± 5.76 years old, participated in this study. The main physical characteristics were as follows: body mass 70.2 ± 5.4 kg, stature 172.7 ± 4.0 cm, and body fat percentage 9.8 ± 3.5%. The sample showed a maximal oxygen consumption (VO_2max_) of 52.3 ± 3.9 mL/kg/min and maximal aerobic power (MAP) of 4.65 ± 0.36 W/kg (Table 1). The athletes compete at an amateur level and accomplish a specific training volume of 14 to18 h per week. Along with their career, they are familiarized and regularly evaluated through incremental protocols, for planning strategies.

The sample is qualified at an intermediate level, considering the aerobic power [27], which places it in a high regional competitive level.

A questionnaire for biographical characterization was also applied. The objectives and the inherent risks of study participation were explained to all the athletes that gave their written informed consent. The study was approved by the Scientific Council of Faculty of Sports and Science and Physical Education of Coimbra University (CE/FCDEF-UC/00132014), and conforms to the ethical standards of this journal [28].

### 2.2. Study Design

In one-week, athletes performed three evaluation tasks on different days. On the morning of the first day (8:00–10:00 a.m.), the athletes came to the laboratory for biographical, anthropometric, and body composition assessment. Then, they underwent an incremental maximal continuous protocol for the determination of VO_2max_ and the ventilatory thresholds. In the following days, and in a randomized order, the athletes performed two field time trials of 20 min at maximum intensity using the same mountain bike (MTB). One in the morning (6:30–10:30 a.m.) and the other in the afternoon (2:30–6:30 p.m.). In the 36 h between each time trial evaluation, the athletes kept a light training load, with a similar standard of living, namely nutrition, hydration, and sleep, and abstained from consuming alcohol in the previous 36 h, and coffee on testing days.

### 2.3. Procedures

#### 2.3.1. Anthropometric and Body Composition Assessment

The anthropometric characterization was done with the athletes barefoot, and wearing cycling shorts. Stature was acceded trough a portable stadiometer (Seca^®^, Hamburg, Germany), body mass was measured with a portable digital scale (Seca^®^, model 770) and body composition assessment was done through bio-impedance (Tanita^®^ BC-601). Prior to the bioimpedance assessment, athletes were warned to avoid drinking water during the previous 2 h and to empty their bladder just before the measurement.

#### 2.3.2. Maximal Incremental Test

A graded maximal continuous protocol was applied to determine the maximal aerobic power VO_2max_ values, and submaximal ventilatory thresholds, as well as, the equivalents of physiological (heart rate), external mechanical power, and lactatemia.

The test protocol was conducted in the laboratory, using a road bicycle and rollers (Tacx^®^ Flow with nine intensity levels), controlling the temperature, cadence, and power (PowerTap^®^). Heart rate (Polar^®^ S 810) was controlled continuously, and ventilatory parameters were assessed trough a portable breath-by-breath gas analyzer (K4b2 da Cosmed^®^). Lactate was measure with a portable enzymatic analyzer (LactatePro^®^).

The data reading and recording were accomplished through a cycle computer (Garmin Edge^®^ 500). The protocol was stopped when compliance with two of the four criteria was verified: lactate higher than or equal to 8 mmol/L; QR higher than or equal to 1.1; increase in VO_2_ less than or equal to 1.5 mL/kg/min despite the increased intensity; heart rate higher than or equal to 95% of maximum heart rate [29,30,31].

The submaximal ventilatory thresholds were determined from the respiratory equivalents of oxygen consumption (V_E_/VO_2_) and carbon dioxide (V_E_/VCO_2_) release. The first ventilatory threshold was assumed the lowest workload coincident with the systematic increase in V_E_/VO_2_ without a concomitant increase in V_E_/VCO_2_. The second ventilatory threshold was considered as the lower workload with concomitant increases in V_E_/VO_2_ and V_E_/VCO_2_ [29].

The potentiometer and gas analyzer were calibrated according to the manufacturer instructions.

An initial period of 15 min of warming up, with less than 100 W and 120 bpm, was applied. After a rest period of 10 min, the graded protocol until exhaustion was performed, with an initial load of 125–130 W, increasing 25 W every 2 min thereafter. The athletes were free to choose the bicycle gearings, cadence, and the levels of intensity on the rollers.

#### 2.3.3. Time Trial

The time trial protocols were conducted in the same ground track, one in the morning and one in the afternoon, lasting 20 min and accomplished at maximal intensity. The second time trial protocol was performed within two days after the first. The stroke rate was chosen freely with athletes using their own mountain bikes.

The athletes performed 15 min warming up below 100 W and at 120 bpm. After the warm-up, a recovery period of 10 min minimum was taken just before the time trial protocol.

Heart rate (Polar^®^ S 810), temperature, stroke rate (cadence) and power (PowerTap^®^) were controlled continuously. The back wheel was equipped with a Continental Race King 2.2 tire (2.4 bars of pressure). Data reading and recording was done through a cycling computer (Garmin Edge^®^ 500). Lactatemia (LactatePro^®^) was assessed just before de time trial and 1, 3, and 5 min after, to determine the maximal lactate value.

### 2.4. Data Analysis

The data mean, minimum, maximum, and standard deviation values are presented as a descriptive statistical analysis. The normality of data distribution was checked by the Shapiro–Wilk test for all the variables. If identified, outlier cases were withdrawn. The comparative analysis was made using a paired sample *T*-test or the equivalent non-parametric Wilcoxon test when normality was not confirmed. Correlation analysis was conducted using the Pearson or Spearman’s Rho tests, whenever justified. The effect size of the mean differences and the interpretation of the correlation coefficient was computed according to Cohen [32,33]. In all statistical analyzes, a significance level of *p* ≤ 0.05 was required. The a priori statistics power was calculated with the software GPower version 3.1.9.6 (Heinrich-Heine-Universität Düsseldorf, Düsseldorf, Germany). The statistical analysis was executed using the SPSS software, version 21 (IBM, Armonk, NY, USA).

## 3. Results

The morning trials were held on average, at 08:35:03 a.m., and the afternoon ones at 4:54:21 p.m. The mean weather temperature was 26.67 ± 2.02 °C in the morning and 33.33 ± 3.75 °C in the afternoon. The results are presented in Table 2.

**Figure 1:**
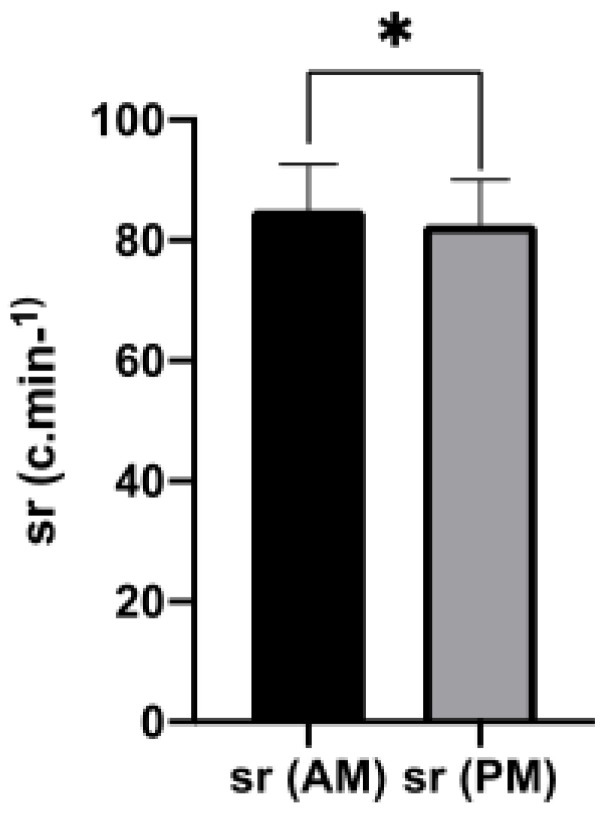
Stroke rate values in the morning (SR_AM) and afternoon (SR_PM) time trials (mean ± SD); * *p* < 0.05.

The comparative analysis between trials revealed the non-existence of differences for most of the variables except in weather temperature and the cadence-stroke rate. The last one showed higher values in the morning (*t* = 2.2; *p* = 0.044) with a medium effect size (*ES* = 0.55) (Table 2, Figure 1).

The weather temperature was higher in the afternoon sessions (*t* = −5.814; *p* < 0.001) with a large effect size (*ES* = 2.21), when compared to the morning sessions.

The maximal heart rate, external mechanical power, and relative external mechanical power, stroke rate, absolute mean power per cycle and relative mean power per cycle showed higher associations between trials (Table 3). The a.m. and p.m. maximal external mechanical power and the initial and final lactatemia did not follow the same trend and did not correlate.

The heart rate values in both trials oscillated between 170 and 171 bpm, which is similar to the corresponding value observed at the maximal aerobic power intensity in the maximum incremental protocol (HR VO_2max_ = 172 ± 9 bpm). Additionally, the HR corresponding to that of the second ventilatory threshold (HR = 156 ± 12 bpm) was different from the mean HR observed in the field protocols.

However, the relative external mechanical power observed in both field trials, oscillated between 3.93 ± 0.42 and 3.98 ± 0.46 W/kg. These values were above the observed values corresponding to those of the second ventilatory threshold determined in the incremental protocol (3.72 ± 0.46 W/kg) but lower than the one registered at maximum relative aerobic power (4.65 ± 0.36 W/kg). In the morning trial, the athletes worked at 85.7% of the maximum relative aerobic power, while in the afternoon, this value was reduced to 84.7%.

## 4. Discussion

The results support a high relationship between morning and afternoon values for most studied variables, which, together with the absence of differences for almost all variables when comparing morning with afternoon trial performance, indicate that there is no significant influence of the time of day in the performed tasks.

The results of this study show that, although within the 20 min of the field protocol athletes can maintain high heart rate values (similar to the observed in VO_2max_ intensity), the same does not happen with mechanical power (which decreases to intensities slightly above the second ventilatory threshold). This result corroborates the studies performed by Grappe [34] who argues that there is no power threshold (in the sense of considering a work interval at a certain intensity that can be maintained over time). There is a maximum working time for each power value. For this author, the produced power is not linear but evolves by levels: the first level, where there is a great difference of values, is between 1 s and 5 min, with the second level between 5 and 60 min. The levels in our maximum oxygen consumption protocol lasted 2 min, while those in the field protocol lasted 20 min. The work is therefore done at a lower power level in the field protocol, justified by its longer duration.

Our study corroborates the conclusions of numerous published papers, where for similar exertions, the circadian rhythm does not influence the heart rate response [15,16,17,21,35].

The observed difference in the morning and afternoon ambient temperatures does not seem to have had an impact in the mean and maximum heart rates observed in the field trials, corroborating several studies where the air temperature, when kept at comfortable levels, did not influence the maximum heart rate [17,23].

Kunorozva et al. [36] presented differences for the relative power output, but in our study, the external mechanical power output was not different between conditions, which is in line with several published work [9,10,11,14,16,17,36].

No differences, in our study, were observed in the initial and final blood lactate values for both protocols. In this domain, the literature is not unanimous: while some studies do not show differences throughout the day [15,37], others observed a higher concentration in the afternoon [11,21,22,38,39].

Moreover, regarding the pedaling cadence—stroke rate, the published studies diverge: Kunorozva et al. [36] did not find changes throughout the day, Moussay et al. [20] reported higher afternoon values, while Brisswalter et al. [40] obtained higher morning values for higher intensities. In accordance with the later, we also observed differences between the two periods, with higher mean values in the morning protocol.

Vogt and collaborators [41] published a study where they concluded that the highest values of power coincide with the highest values of pedaling cadence–stroke rate. Lucia et al. [42] came to a similar conclusion, stating that the low stroke rates are less efficient. Our study is in accordance with those studies, showing that the higher mean power values are achieved with higher stroke rate values.

The variation of the bicycle gearings is essentially related to the athlete’s need to keep within the range of cadence considered more comfortable for the power produced at the time [43].

It is interesting to discuss the reason for the differences in morning and afternoon stroke rate mean values. This may be due to several factors, such as increased central body temperature, a musculoskeletal origin factor, or improvement of neuromuscular efficiency, among others. The increase of central body temperature, for example, may lead to decreased muscle viscosity and improved speed of conduction of nerve stimuli, as well as improvements in range of motion and muscle coordination [40]. Although we did not control the body temperature, a study, using thermography, concluded that skin temperature may be associated with postural differences [44], which may also justify this change in stroke rate, since different ambient temperatures were registered for the morning and afternoon time trials.

An adaptation of the motor patterns may have occurred, which caused an adjustment of cadence for the power produced during that time interval. However, a second alternative question arises: what is the reason why our athletes did not keep the mean stroke rate registered in the morning and increased the power values in the afternoon period? It is not easy to find an explanation for this question, but we believe that this may be due to the power limit that each athlete can produce in 20 min at maximum intensity, with this limit reached in both the morning and afternoon time trials. Our hypothesis is based on the analysis of the heart rate intensity level, when compared to that observed in the VO_2max_ incremental protocol. In accordance with Grappe [34], we believe that they have limited themselves, in the afternoon period, to adjust the motor standards that would allow them to work at the appropriate stroke rate since this limited the intensity of power production.

## 5. Conclusions

In conclusion, we have shown that the results obtained in the protocols performed at different times of the day were similar, with no significant chronobiological influence. The exception was the stroke rate.

The practical implication of this result is that, in training and competitions of long-distance MTB, it is not justifiable to plan differently for the morning or afternoon periods, since no significant circadian effects were observed in the performance of athletes. This conclusion should be applied only to athletes of an intermediate level. The repercussion in a sample made up of elite athletes, is unknown. Factors such as the quality of the opponents, the intrinsic or extrinsic motivation, the competition characteristics, qualifying trials or finals, could assume relevance, determining the results. However, due to the fact that they are outside the scope of this study, the possible adaptations of the body to the usual training and competition hours, and the effect of the ambient temperature on the central body temperature, were not discussed.

## Figures and Tables

**Table 1:** Descriptive and statistical (minimum, maximum, mean and standard deviation (SD) values) values of the analyzed variables in the incremental maximal continuous protocol.

Variables	VO_2max_			1st Ventilatory Threshold			2nd Ventilatory Threshold		
	Mean ± SD	Max	Min	Mean ± SD	Max	Min	Mean ± SD	Max	Min
Mean power (W)	324.9 ± 20.16	353	303	178 ± 20.41	203	153	259.3 ± 25	303	203
Relative mean power (W/kg)	4.65 ± 0.36	5.37	3.79	2.55 ± 0.37	3.17	1.91	3.72 ± 0.46	4.43	2.81
Heart rate (bpm)	172 ± 9	188	159	133 ± 12	154	119	156 ± 12	175	137
VO_2max_ (mL/min)	3658.6 ± 213.3	4108	3331	2345.9 ± 193.6	2693	1999	3119.6 ± 172.4	3383	2687
VO_2max_ (mL/kg/min)	52.3 ± 3.9	59.3	43.2	33.6 ± 3.9	40.6	27.3	44.7 ± 4.2	51.3	38.1
QR	1.16 ± 0.07	1.3	1.08	0.94 ± 0.03	1.06	0.9	1.03 ± 0.05	1.12	0.94

**Table 2:** Descriptive statistics (minimum, maximum, mean, and standard deviation (SD) values) values for the analyzed variables during the 20 min maximum protocol time trial on the mountain bike.

Morning					Afternoon		
Variables	Mean ± SD	Minimum	Maximum	Mean ± SD	Minimum	Maximum	*p*
Starting time	08:35:03 ± 00:29:52	07:20:43	09:39:55	16:54:21 ± 00:50:27	14:38:57	18:10:29	---
Temperature (°C)	26.67 ± 2.02	22	30	33.33 ± 3.75	23	38	<0.001 **
Lactate initial (mmol/L)	3.51 ± 1.51	1.7	7	3.47 ± 1.78	1.6	8.4	0.921
Mean heart rate (bpm)	170 ± 9	160	188	171 ± 9	155	187	0.510
Maximal heart rate (bpm)	179 ± 9	168	195	179 ± 9	164	194	0.921
Mean power (W)	278.56 ± 35.61	231	356	275.25 ± 31.94	239	359	0.303
Relative Mean power (W/kg)	3.98 ± 0.46	3.30	4.67	3.93 ± 0.42	3.19	4.71	0.625
Maximal power (W)	767.19 ± 134.59	593	1024	736.00 ± 101.63	578	911	0.761
Stroke rate (Cycles/min)	85.06 ± 7.58	70	95	82.63 ± 7.41	66	94	0.044 ^★^
Mean Power per Cycle (W/Cycle/min)	3.3 ± 0.503	2.62	4.51	3.35 ± 0.434	2.74	4.49	0.429
Relative Mean power per cycle (W/kg/Cycle/min)	0.047 ± 0.007	0.033	0.059	0.047 ± 0.007	0.041	0.061	0.430
Final lactate concentration (mmol/L)	12.82 ± 4.46	5.8	21.2	12.90 ± 6.11	3.6	23	0.718

^★^*p* < 0.05; ** *p* < 0.001.

**Table 3:** Correlations (Pearson) between the physiological, mechanical, and kinematic variables observed in the morning and afternoon time trials.

Variables	Correlation (Pearson)
Mean heart rate (HR)	0.871 **
Maximum heart rate (HRmax)	0.845 **
Mean external mechanical power (PO)	0.938 **
Relative mean external mechanical power (PO/Body mass)	0.933 **
Stroke rate	0.825 **
Mean Power per Cycle	0.801 **
Relative Mean power per cycle	0.884 **

** *p* < 0.001.
